# Identification of a Novel Alternative Splicing Variant of *VvPMA1* in Grape Root under Salinity

**DOI:** 10.3389/fpls.2017.00605

**Published:** 2017-04-21

**Authors:** Ning Han, Xing-Long Ji, Yuan-Peng Du, Xi He, Xin-Jie Zhao, Heng Zhai

**Affiliations:** ^1^Department of Pomology, College of Horticulture Science and Engineering, Shandong Agricultural UniversityTaian, China; ^2^Shandong Provincial Key Laboratory of Microbial Engineering, School of Biologic Engineering, Qi Lu University of TechnologyJinan, China

**Keywords:** grape root, alternative splicing, N-terminal regulation of PM H^+^-ATPase, expression profile, salt stress

## Abstract

It has been well-demonstrated that the control of plasma membrane H^+^-ATPase (PM H^+^-ATPase) activity is important to plant salt tolerance. This study found a significant increase in PM H^+^-ATPase (PMA) activity in grape root exposed to NaCl. Furthermore, 7 *Vitis vinifera* PM H^+^-ATPase genes (*VvPMAs*) were identified within the grape genome and the expression response of these *VvPMAs* in grape root under salinity was analyzed. Two *VvPMAs* (*VvPMA1* and *VvPMA3*) were expressed more strongly in roots than the other five *VvPMAs*. Moreover, roots exhibited diverse patterns of gene expression of *VvPMA1* and *VvPMA3* responses to salt stress. Interestingly, two transcripts of *VvPMA1*, which were created through alternative splicing (AS), were discovered and isolated from salt stressed root. Comparing the two *VvPMA1* cDNA sequences (designated *VvPMA1*α and *VvPMA1*β) with the genomic sequence revealed that the second intron was retained in the *VvPMA1*β cDNA. This intron retention was predicted to generate a novel VvPMA1 through N-terminal truncation because of a 5′- terminal frame shift. Yeast complementation assays of the two splice variants showed that *VvPMA1*β could enhance the ability to complement *Saccharomyces cerevisiae* deficient in PM H^+^-ATPase activity. In addition, the expression profiles of *VvPMA1*α and *VvPMA1*β differed under salinity. Our data suggests that through AS, the N-terminal length of VvPMA1 may be regulated to accurately modulate PM H^+^-ATPase activity of grape root in salt stress.

## Introduction

Salinity is one of the most important abiotic stresses limiting crop productivity, and the amount of land affected by salinity is increasing (Wakeel et al., 2010). Salt stress has been found to affect the activity of the plasma membrane H^+^-ATPase (PM H^+^-ATPase) in plant (Janicka-Russak et al., 2013). The PM H^+^-ATPase is part of a large family of ion transport proteins termed P-type ATPases, which are responsible for generating the electrochemical proton motive force across the plasma membrane of plant and fungal cells. The H^+^-ATPase is thus essential for energizing the plasma membrane (Palmgren, 2001; Arango et al., 2003) and is involved in many physiological roles, such as solute transport into the cell, cell pH homeostasis, and elongation (Duby and Boutry, 2009). Moreover, PM H^+^-ATPas provides the driving force for potassium retention and uptake through voltage-gated channels and for Na^+^ exclusion via Na^+^/H^+^ exchangers during salt stress (Kabała and Janicka-Russak, 2012; Hasegawa, 2013). Both of these traits are central to plant salinity tolerance. Therefore, the control of PM H^+^-ATPase activity is important to plant salt tolerance (Gévaudant et al., 2007; Janicka-Russak et al., 2013).

In plants, multiple mechanisms have evolved to regulate H^+^-ATPase expression and activity (Haruta et al., 2015; Falhof et al., 2016). At the posttranslational level, PM H^+^-ATPase activity is controlled by a C-terminal auto-inhibitory domain of about 100 residues (Piette et al., 2011). It is also well-established that protein kinase-mediated phosphorylation of the penultimate threonine can release the auto-inhibition of the C-terminal region, resulting in a hyperactive enzyme, with increased catalytic activity (Lecchi et al., 2008). In addition, other phosphorylated residues of the C-terminal region have been identified, and some have been shown to reduce or increase enzyme activity (Duby et al., 2009; Hsu et al., 2009). Unfortunately, the specific kinases that catalyze this reaction are still uncertain. Recently, the N-terminal region has also been proposed to be involved in enzyme auto-inhibition. Complete removal of the N terminus of the PM H^+^-ATPase *in vitro* results in activation of the PM H^+^-ATPase (Ekberg et al., 2010). However, as the enzyme hyperactivity caused by phosphorylation of the N-terminal region seems an ineffective regulatory mechanism, until now, the mechanisms though which regulation of the N-terminal region of PM H^+^-ATPase is achieved *in vivo* were unclear (Rudashevskaya et al., 2012).

Previous studies have shown that under saline conditions, fast posttranslational modification in the C-terminal region of PM H^+^-ATPase can occur (Kalampanayil and Wimmers, 2001; Janicka-Russak and Kłobus, 2007). In addition to posttranslational modification, increases in PM H^+^-ATPase transcription in response to NaCl exposure has been reported (Zhang et al., 1999; Chen et al., 2007; Sahu and Shaw, 2009).

In most plant species, the PM H^+^-ATPase is encoded by a multigene family. Extensive searching of cDNA and genomic libraries from *Nicotiana plumbaginifolia* results in the identification of nine PM H^+^-ATPase genes (Perez et al., 1992; Moriau et al., 1993; Oufattole et al., 2000). Complete sequencing of the Arabidopsis genome uncovered 12 genes (Palmgren, 2001). The existence of multiple isoforms of the enzyme might suggest considerable overlap of isoform expression in many cell types and tissues Gévaudant et al., 2001. Nonetheless, isoform diversity may also be related to cellular differentiation with individual isoforms exhibiting tissue, developmental, and environmental-specific expression and having slightly different biochemical and regulatory properties (Palmgren, 2001; Arango et al., 2003). The functional significance of the multiple isoforms of PM H^+^-ATPase, such as the possibility of their role in salt stress tolerance, is not well-understood, although PM H^+^-ATPase does respond to salt stress. For example, in rice cultivars, salt treatment induced expression of a new isoform of the PM H^+^ATPase gene (Sahu and Shaw, 2009).

*Vitis vinifera* (*V. vinifera*) is one of the world's oldest fruits and nowadays grows worldwide. Grapevine is adapted to semiarid environments, where drought and salinity are common problems. Grapevine is considered moderately tolerant to salt stress, and this moderate tolerance has been mainly attributed to salt exclusion or to restriction of toxic ions in the root system (Walker et al., 2002; Ma et al., 2015), which needs PM H^+^-ATPase of root to provide driving force. Up to now, research on PM H^+^-ATPases has mainly focused on annual herbs, while there is little information about the regulation of PM H^+^-ATPase activity in perennial woody plants, such as *V. vinifera* under saline conditions. Here the PM H^+^-ATPase isoforms within the grape genome (Jaillon et al., 2007) were identified and the effect of salinity on the expression of root specific isoforms was analyzed in grapevine.

## Methods

### Plant materials and growth conditions

The table-grape cultivar Crimson seedless (C133–199 × Emperor) was used in this study. The sterilized seedlings were grown on 1/2 MS solid medium (Murashige and Skoog, 1962) in glass bottles (9 cm height × 6 cm diameter) at a 25/20°C growth room with white light illumination (120 μmol photons per m^2^ s^1^) under a 16/8 h light/dark photocycle. Shoot tips with a minimum of one bud were used to subculture every month. After 30 days, uniform plants were selected as the test material and were transferred into Hoagland solution (2 mM MgSO_4_, 5 mM Ca(NO_3_)_2_, 5 mM KNO_3_, 1 mM KH_2_PO_4_, and microelements contained 0.2 mM Fe-EDTA, 2 × 10^−3^ mM MnCl_2_, 2.5 × 10^−4^ mM H_3_BO_3_, 5 × 10^−4^ mM CuSO_4_, 2 × 10^−3^ mM ZnSO_4_ and 5 × 10^−4^ mM H_2_MoO_4_) for 7 days. The growth conditions were kept the same as above. The test seedlings were treated with 50 mM NaCl in Hoagland solution for 3, 6, 12, 24, 48, and 72 h, respectively. And the seedlings grown in Hoagland alone were used as the control (0 h). Samples were then harvested at the same time in the same day to minimize diurnal effects wherever possible, then frozen in liquid nitrogen and stored at −80°C.

### Membrane vesicle isolation

Plasma membrane-enriched vesicles were isolated by discontinuous sucrose gradient centrifugation, according to the method of Wang et al. (2001) and Mandala and Taiz (1985) with modifications. Roots were washed with cold deionized water and homogenized in extraction medium containing 50 mM Tricine-Tris (pH 7.8), 3 mM EGTA, 3 mM MgSO_4_, 0.5% (V/V) PVP, 2 mM DTT, 0.2 mM PMSF, 5% (V/V) glycerol, and mannitol, which produced the same osmotic potential as within leaves. Two milliliters of the medium was used for each 2 g of fresh material. The homogenate was filtered through four layers of cheesecloth and centrifuged at 10,000 g for 20 min (Beckman JA-20). The supernatant was loaded on a 0%: 36%: 45% (W/W) sucrose gradient solution (5 mM HEPES adjusted to pH 7.5 with Tris, and 1 mM DTT) and centrifuged at 100,000 g for 2 h (Beckman SW40Ti). The plasma membrane-enriched vesicles were located at the 36%: 45% (W/W) sucrose interface. These vesicles were carefully collected and diluted 3–4-fold with dilution buffer (3 mM MgSO_4_, 10 mM HEPES–Tris pH 7.5, 1 mM DTT) and centrifuged at 100,000 g for 30 min (Beckman type 65). The pellets were suspended in a storage buffer (40% (V/V) glycerol, 2 mM DTT, 10 mM HEPES, adjusted to pH 7.0 with KOH).

### Measurement of protein content

Protein content was determined using bovine serum album as a protein standard according to the Bradford method (Bradford, 1976).

#### ATPase assays

PM H^+^-ATPase hydrolytic activity was calculated from the amount of inorganic phosphate released in the absence and presence of 0.1 mM Na_3_VO_4_. The enzyme reaction was run at 37°C for 30 min with 20 μg of membrane protein in an assay medium containing 30 mM Tris/Mes pH 6.5, 0.1 mM (NH_4_)_2_MoO_4_, 1 mM NaN_3_, 1 mM MgSO_4_, 0.03% (V/V) TritionX-100, 50 mM KCl, and 3 mM ATPNa_2_. Inorganic phosphate was assayed by using the method of Lin and Morales (1977).

### Genome wide search and identification of VvPMAs

The highly conserved domain “GDGVNDAPALK” was defined as the probe sequence according to the alignment results of published PM H^+^-ATPase amino acid sequences in plants (Palmgren, 2001). The non-redundant protein database of *V. vinifera* was retrieved through blastp on the NCBI website (National Center for Biotechnology Information, www.ncbi.nlm.nih.gov). Then the PM H^+^-ATPase functional domains of these candidate proteins were predicted using Pfam software (http://pfam.sanger.ac.uk; Finn et al., 2010). The corresponding gene sequences and cDNA sequences of these domain-verified PM H^+^-ATPases were acquired using accession numbers.

### Phylogenetic tree construction and multiple sequence alignment of VvPMAs

The phylogenetic tree was constructed using MEGA 5.0 software and making comparisons between candidate VvPMAs and previously identified AHA proteins from *A. thaliana*.

### Total RNA isolation from grape

Total grape RNA was extracted using the CTAB method as previously described (Gambino et al., 2008), with a few modifications. Root samples (200 mg) were ground with mortar and pestle under liquid nitrogen. The powder was transferred to an RNase-free tube containing 2 mL CTAB extracting buffer (2% CTAB, 2.5% PVP-30, 2 M NaCl, 100 mM Tris-HCl (pH 8.0), 25 mM EDTA, and 2% 2-mercaptoethanol) and mixed. Then the samples were incubated at 65°C for 10 min. After centrifugation (4°C, 10, 000 *g*, 10 min), the supernatant was then transferred to an equal volume of extraction cocktail [chloroform/isoamyl alcohol (24/1)] in order to extract the RNA. The mixture was centrifuged at 10, 000 *g* for 10 min, after which, the supernatant was mixed with LiCl (3 M) and stored at −20°C for 12 h. After centrifugation (4°C, 10, 000 *g*, 20 min), the pellets were suspended in SSTE buffer [1 M NaCl, 1% SDS, 1 mM EDTA, and 10 mM Tris-HCl (pH 8.0)]. A chloroform/isoamyl alcohol (24/1) mixture was added, and then the samples were centrifuged at 10, 000 g for 10 min. The supernatant was transferred to a new RNase-free tube and mixed with 0.7 volume isopropanol. After centrifugation at 10,000 g for 20 min, the pellets were washed with 1 ml 75% alcohol, dried, and resuspended in RNase free water. The total RNA was quantified with a NanoDrop 2000C spectrophotometer (Thermo Scientific, USA) at wavelengths of 230, 260, and 280 nm and the 260/280 nm ratio was determined. RNA integrity was confirmed by running samples through 1% agarose gels. After that samples were stored at −80°C.

### Quantitative RT-PCR (qRT-PCR) for analysis of gene expression

First-strand cDNA was synthesized from approximately 3 μg of total RNA using the PrimeScript™ RT reagent Kit with gDNA Eraser (TaKaRa, Dalian, China) according to the manufacturer's instructions. Quantitative PCR was carried out on a real-time PCR system (Bio-Rad iQ5) using the SYBR Green Kit (TaKaRa, Dalian, China). Thermal cycling conditions were 95°C for 10 min followed by 95°C for 10 s, 56°C for 10 s for 40 cycles, and a melting cycle was then performed from 65° to 95°C. A house-keeping gene *actin* was used as an internal control for qPCR (Reid et al., 2006), and it was previously used as a reliable internal reference gene in grape under salt stress (Ma et al., 2015). *VvPMAs* transcripts were quantified after normalization to *actin*. Results are reported as 2^−ΔCT^ or 2^−ΔΔCT^, where ΔCT is the number of PCR cycles required for the log phase of amplification for the experimental gene minus the same measure for *actin* and ΔΔCT is the ΔCT of treatment minus the ΔCT of control (0 h) for the same gene (Livak and Schmittgen, 2001). Primer sequences used for the qPCR analyses were shown in Supplementary Table 1.

### Cloning and sequencing of the PM H^+^-ATPases isoform *VvPMA1*

RNA was extracted from the root of a Crimson seedless grape plant and cDNA synthesis was carried out as described above. The full-length cDNA encoding *VvPMA1* was isolated through PCR using *Pfu* DNA polymerase (TaKaRa, Dalian, China) and the primers listed in Supplementary Table 2. The generated PCR fragment was purified and directly inserted into the pEASY-Blunt vector following the manufacturer's instructions (Transgen Biotech. Co., China). The plasmids were transformed into *E. coli* DH5α cells. Finally, the 9 recombinant plasmids were prepared and then sequenced, respectively.

### Expression of PM H^+^-ATPases in *Saccharomyces cerevisiae*

Plasmid pMP625 containing the promoter and terminator of *PMA1* (yeast endogenous PM H^+^-ATPase) was kindly provided by Dr. Palmgren (University of Copenhagen, Denmark). The full-length coding regions of *VvPMA1*α and *VvPMA1*β were amplified from pEASY-Blunt-*VvPMA1*α and pEASY-Blunt-*VvPMA1*β using the primers *VvPMA1*α-P1, 5′CTCGAGATGGGAGGCGACAAATCC 3′, which includes an underlined *Xho*I site, and *VvPMA1-*P2, 5′ GGGCCCTCATACTGTGTAATGCTGTTGGATT 3′, with an underlined *Apa*I site; *VvPMA1*β-P1, 5′CTCGAGATGCGCATTTCATTGAATT 3′ and *VvPMA1-*P2, 5′ GGGCCCTCATACTGTGTAATGCTGTTGGATT 3′. The PCR products were subcloned into *Xho*I and ApaI sites of pMP625 to generate plasmid pMP625-*VvPMA1*α and pMP625-*VvPMA1*β. All PCR amplifications were carried out using *Pfu* DNA polymerase, which exhibits the lowest error rate of any thermostable DNA polymerase, and all constructs were sequenced to confirm their identity.

### Yeast strains and culture conditions

The yeast strain RS72 (also gifted from Dr. Palmgren) was cultured as described previously (Ekberg et al., 2010). In RS72 (*MATa ade1–100 his4–519 leu2–3, 112*), the natural constitutive promoter of the *PMA1* has been replaced by the galactose-dependent *GAL1* promoter. As *PMA1* is essential for yeast growth, RS72 is only able to grow on galactose-containing medium. Using this strain, plasmid-borne plants' PM H^+^-ATPases under the control of the constitutive *PMA1* promoter can be tested for their ability to rescue *pma1* mutants on glucose-containing medium.

Yeast was made competent for plasmid uptake by treatment with lithium acetate and polyethyleneglycol according to Gietz et al. (1995). Positive transformants were selected on synthetic medium containing 2% (w/v) galactose, 0.7% (w/v) yeast nitrogen base without amino acids (Difco), 0.2 mM adenine, 0.4 mM histidine, without leucine after 3 days of growth at 30°C. And transformants were grown for 3 days at 30°C in liquid synthetic medium containing 2% galactose. Then, 5 ml transformed yeast cells were transferred to solid at two different concentrations (OD_600_ 0.1 and OD_600_ 0.01) or 20 ml liquid synthetic medium at OD_600_ 0.01 containing either 2% galactose at pH 5.5 or 2% glucose at pH-values of 5.5, 4.5, and 3.5. The media were buffered with 50 mM succinic acid adjusted to various pH-values with Tris. Growth was recorded after incubation for 3 days at 30°C. Each experiment was replicated independently three times.

### Statistical analysis

Statistical analyses were performed using the software SigmaPlot 10.0 (Systat Software, Inc). Values were expressed as mean ± SD. All comparisons were done using Student's *t*-test for independent samples. In all cases, the confidence coefficient was set at 0.05.

## Results

### Effect of NaCl on PM H^+^-ATPase activity

**Figure 1:**
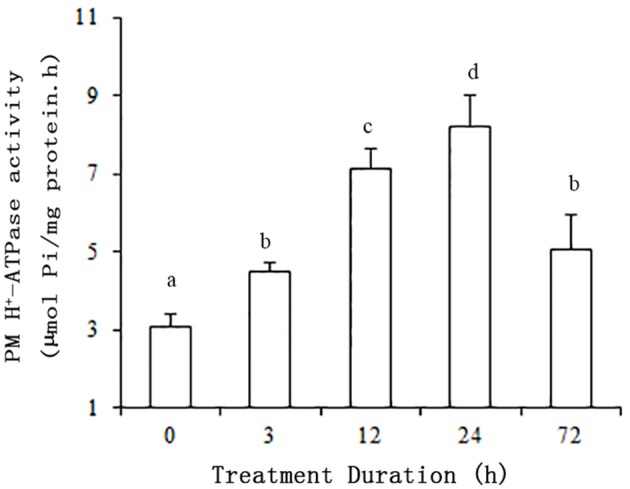
**Effect of NaCl on the hydrolytic activity of PM H^**+**^-ATPase in grape root**. The plasma membranes were isolated from roots treated with 50 mM NaCl for 0, 3, 12, 24, and 72 h. The values are means (*n* = 9) of three independent experiments ± *SD*. Values with the same letter are not significantly different (*P* > 0.05).

The plants were subjected to salt stress (50 mM NaCl) for 0, 3, 12, 24, or 72 h. ATP hydrolytic activity in PM vesicles obtained from plant roots increased under the NaCl treatments. Moreover, the enzyme activity was up regulated within 3 h of treatment (*P* < 0.01). The maximum increase in enzyme activity resulted in an activity about 2.7-fold greater than that of the control at 24 h. Although the enzyme activity decreased between 24 and 72 h, the activity level remained above the control activity level (*P* < 0.01) (Figure 1).

### Identification of *VvPMAs* through genome-wide analysis

To explore the prevailing isoforms of PM H^+^-ATPase genes in grape root treated with salt stress, the *VvPMA* genes were screened first from genome of the near-homozygous PN40024 genotype of *V. vinifera cv* Pinot Noir. According to the retrieved candidate VvPMA proteins and Pfam software verification, we obtained putative 14 VvPMAs *in silico*. Subsequently, we eliminated overlapping protein sequences and short fragments based on the functional domain sequence. Finally, a total of 7 candidate genes were identified as possible grape PM H^+^-ATPase genes (Table 1).

**Table 1:** **Grapevine PM H^**+**^-ATPase genes identified in the PN40024 12 × V1 prediction**.

**Proposed nomenclature**	**PN40024 12 × v1 ID**	**Refseq**	**ORF (aa)**	**Coordinates**
*VvPMA1*	VIT_00029244001	XM_002267465.2	954	Chr11: 8105024.18111386
*VvPMA2*	VIT_00018265001	XM_003633847.2	968	Chr15: 2287766.12296627
*VvPMA3*	VIT_00035525001	XM_002282583.2	954	Chr4: 2012079.2019796
*VvPMA4*	VIT_00032599001	XM_002282227.3	955	Chr14: 8745554.28754977
*VvPMA5*	VIT_00001052001	XM_002263205.2	952	Chr11: 7097145.7102056
*VvPMA6*	VIT_00008074001	XM_002270308.3	956	Chr17: 6042714.6050674
*VvPMA7*	VIT_00016862001	XM_002280165.2	950	Chr9: 2069774.2076128

**Figure 2:**
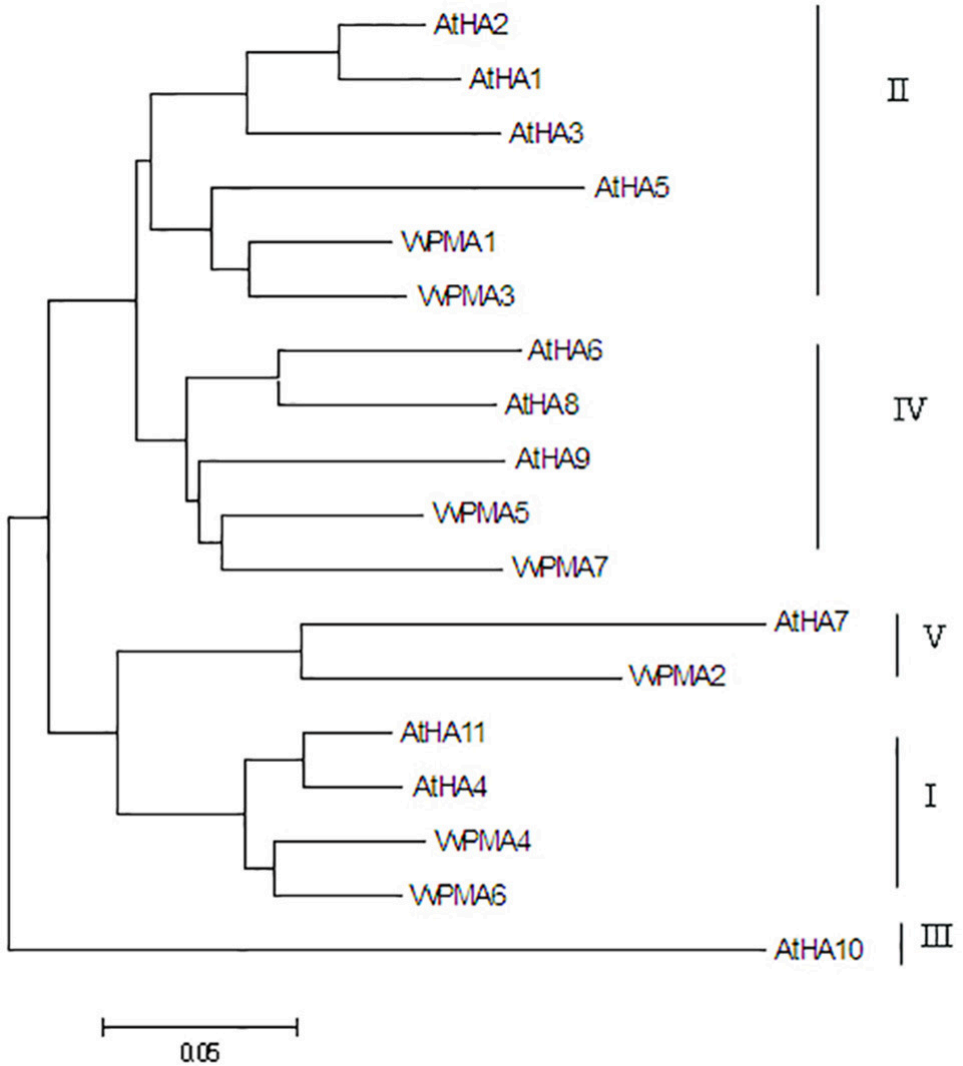
**Phylogenetic tree of the PM H^**+**^-ATPase proteins from ***V. vinifera*** (VvPMA1-7) and ***A. thaliana*** (AtHA1-11)**. The phylogenetic tree was created using the MEGA 5.0 software. Roman numerals designated the subfamilies.

In order to determine the evolutionary relatedness among the PM H^+^-ATPases from grape and Arabidopsis, a phylogenetic tree was constructed using MEGA 5.0 software and the deduced amino acid sequences. The phylogenetic analysis showed PM H^+^-ATPase protein from each of the two species to fall within the same clades with a high degree of similarity. Additionally, VvPMAs were classified into four subfamilies according to Arango et al. (2003; Figure 2).

### Expression of *VvPMAs* in grape root

**Figure 3:**
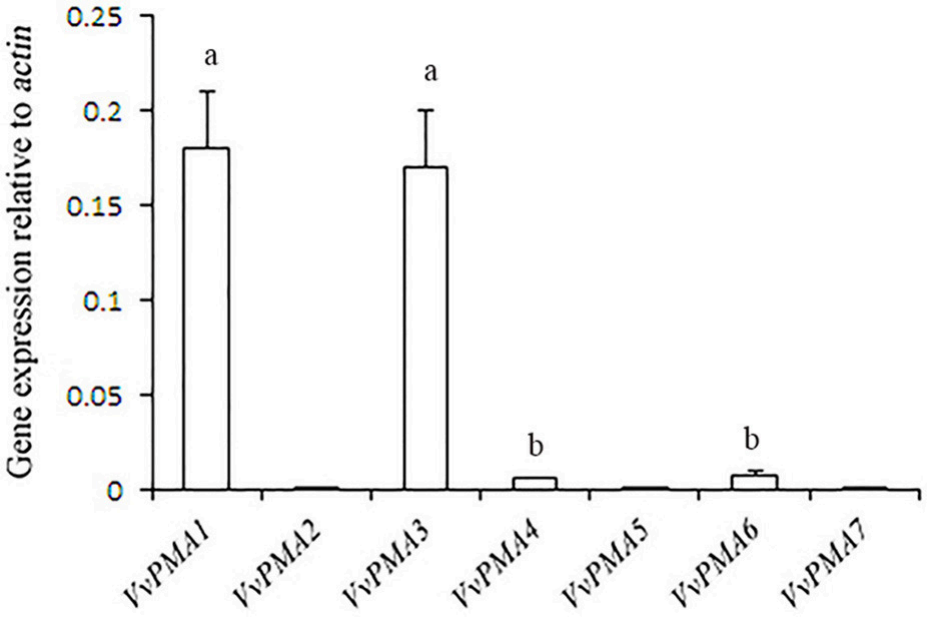
**The relative expression levels of the different ***VvPMA*** isoforms in grape root**. *Vvactin* was used as an internal standard. The data were analyzed according to 2^−ΔCT^ method. The values are means ± *SD* (*n* = 5). Values with the same letter are not significantly different (*P* > 0.05).

In seedling roots grown under normal conditions, expression of *VvPMA1* and *VvPMA3* was markedly higher than *VvPMA4* and *VvPMA6* expression (*P* < 0.01), but expression levels did not significantly differ between *VvPMA1* and *VvPMA3* (*P* > 0.05). Additionally, although expression of *VvPMA2, VvPMA5*, and *VvPMA7* was found in other tissues (Supplementary Figure 1), their expression was undetectable in roots using either of two different primer pairs (Figure 3).

**Figure 4:**
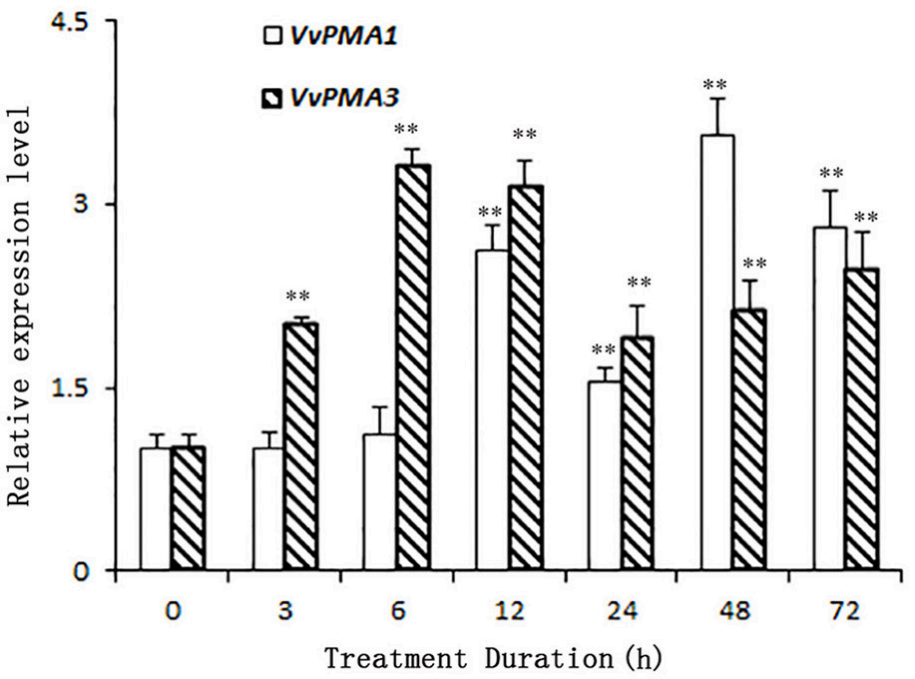
**The relative expression levels of ***VvPMA1*** and ***VvPMA3*** in grape root under 50 mM NaCl stress for 0–72 h**. *Vvactin* was used as an internal standard. The data were analyzed according to 2^−ΔΔCT^ method. The values are means ± *SD* (*n* = 5). ** are significantly different from 0 h at *P* < 0.01.

As the predominant genes expressed in root, *VvPMA1* and *VvPMA3* were greatly up-regulated under 50 mM NaCl stress (Figure 4). Moreover, *VvPMA1* expression was not up-regulated significantly until 12 h of treatment, and then reached its maximum level at 48 h, when it was as much as 3.6 times greater than that of the control (*P* < 0.01; Figure 4). Expression remained greater than control levels until 72 h of treatment. For *VvPMA3*, expression was highest at 6 h of treatment, when it was 3.3-fold greater than at 0 h. After that, expression reduced, but remained above 0 h levels until 72 h of treatment (*P* < 0.01; Figure 4).

### Cloning and alternative splicing of *VvPMA1*

**Figure 5:**
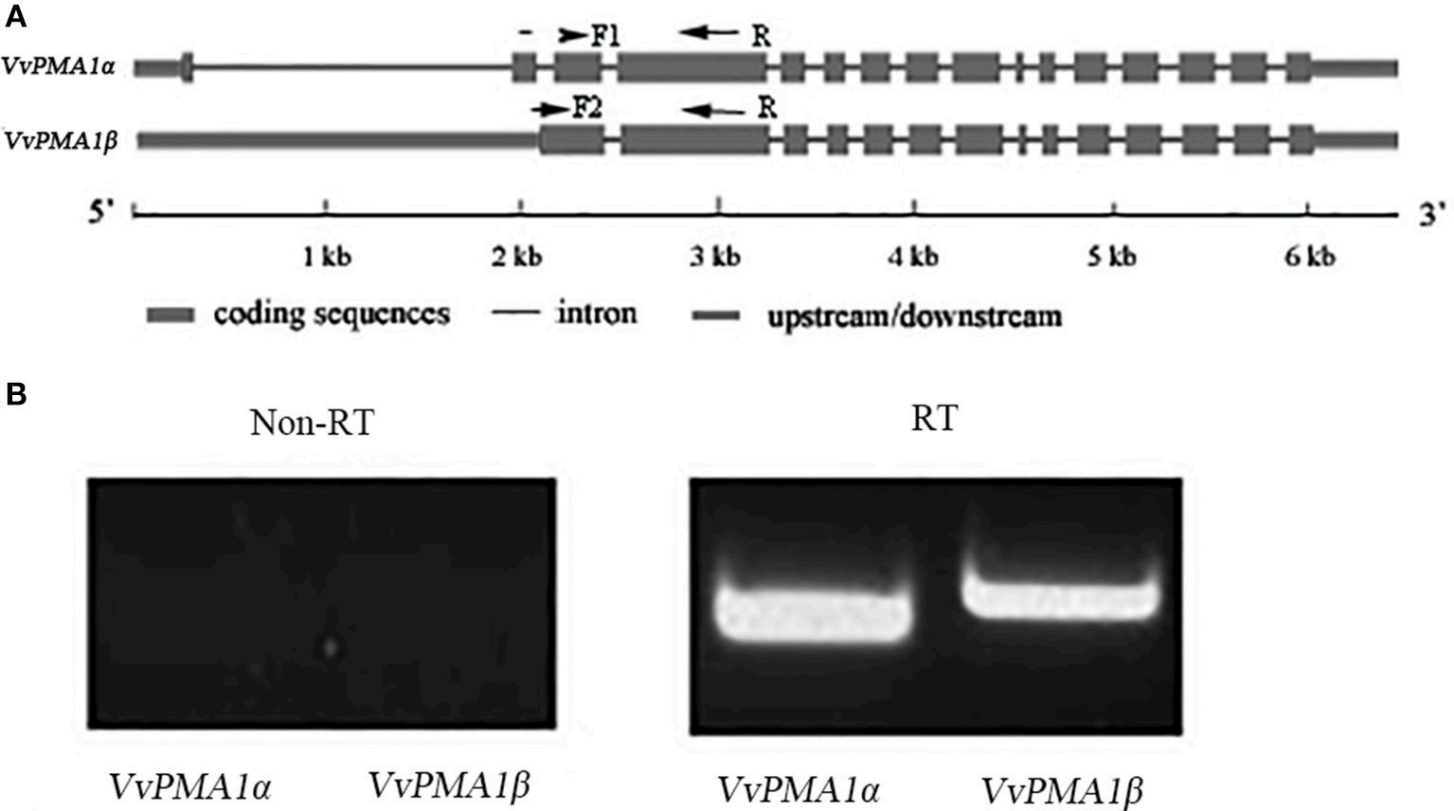
*****VvPMA1*** alternative splicing events in grape root. (A)** Genomic structure of *VvPMA1* and the alternatively spliced variant. F1 and F2, forward primers; R, reverse primer. **(B)** Detection of alternatively spliced *VvPMA1* by RT–PCR in grape root in 50 mM NaCl stress for 3 h.

To study the function of *VvPMA1*, the full-length cDNA sequence of *VvPMA1* was cloned and sequenced through reverse transcription PCR (RT-PCR). Interestingly, two different cDNA sequences of *VvPMA1* were obtained. Comparison of the two *VvPMA1* cDNA sequences (designated *VvPMA1*α and *VvPMA1*β) with the genomic sequence revealed that the second intron (93 bp) was retained in the *VvPMA1*β cDNA (Figure 5A, Supplementary Figure 2). Furthermore, RT-PCR using *VvPMA1*α and *VvPMA1*β specific primers (Figure 5A, Supplementary Table 1) detected both transcripts, respectively after 3 h of 50 mM NaCl stress (Figure 5B), proving that AS of *VvPMA1* really occur.

The predicted VvPMA1α and VvPMA1β proteins had identical C domains, whereas a frame shift resulted in a loss of 35 amino acids at the N-terminus of VvPMA1β (Supplementary Figure 3).

### Complementation of yeast mutants by two *VvPMA1* splice variants

**Figure 6:**
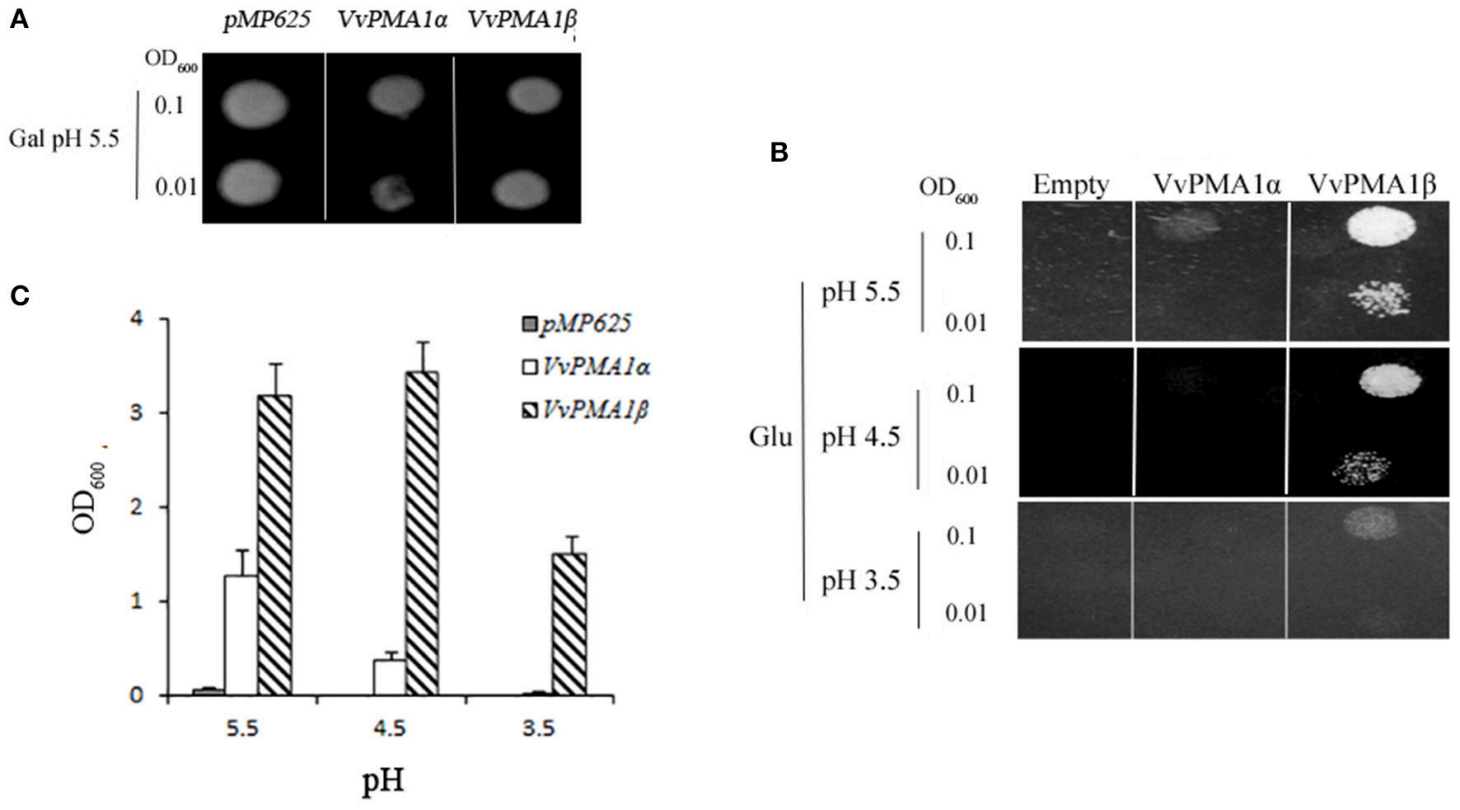
**Activities of ***VvPMA1*** spliced variants in a yeast complementation assay**. Drop test for the growth of yeast strains on galactose **(A)** and glucose **(B)** solid medium or in glucose liquid medium **(C)**. In the yeast strain RS72, the endogenous yeast PM H^+^-ATPase (PMA1p) has been placed under the control of a galactose promoter. The introduced, plasmid-borne *VvPMA1* are under the control of the constitutive *PMA1* promoter. Yeast growth on glucose is therefore dependent on a functional plasmid-borne VvPMA1. Transformed yeast cells, were spotted on either galactose-containing solid media (*Gal*) at pH 5.5 **(A)** or glucose-containing solid media (*Glu*) at different pH-values **(B)** at two different concentrations (OD_600_ = 0.1 and OD_600_ = 0.01). And same inoculated into glucose-containing liquid media at OD_600_ = 0.01 **(C)**. Growth was recorded after 3–4 days at 30°C. The values are means ± *SD* (*n* = 3).

To investigate whether both *VvPMA1*α and *VvPMA1*β encode functional proton pumps and test for their ability to complement *pma1*, we expressed them in mutant yeast RS72. The results were shown in Figure 6. The negative control (the strain MP625 with the yeast *PMA1* under the GAL1 promoter) grew well in galactose medium only (Figure 6A). The strains with *VvPMA1*α and *VvPMA1*β both supported yeast growth on either glucose solid or liquid medium (Figures 6B,C), however, at pH 5.5 and more acidic pH levels, *VvPMA1*β increased yeast growth by a greater amount than *VvPMA1*α did (Figures 6B,C).

### Gene expression of the two *VvPMA1* splice variants in grape root under salt stress

**Figure 7:**
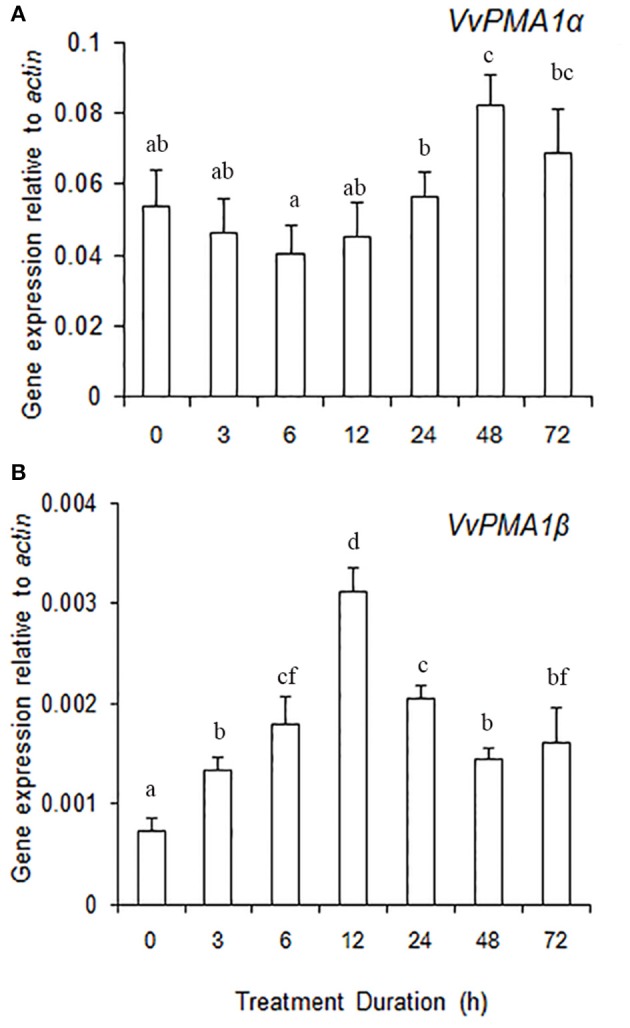
**The relative expression levels of ***VvPMA1*** spliced variants, ***VvPMA1***α (A)** and *VvPMA1*β **(B)**, in grape root under 50 mM NaCl stress for 0–72 h. *Vvactin* was used as an internal standard. The data were analyzed according to 2^−ΔCT^ method. The values are means ± *SD* (*n* = 5). Values with the same letter are not significantly different (*P* > 0.05).

To ascertain the regulatory mechanisms controlling the two *VvPMA1* splice variants under salt stress, the expression levels of the two variants were examined in root under salt treatments. The results showed expression of *VvPMA1*α to be much higher than *VvPMA1*β expression in salt and fresh water treated roots (Figures 7A,B). However, *VvPMA1*α expression had not too much change until 48 h of treatment (Figure 7A), whereas, *VvPMA1*β expression increased rapidly and peaked at 12 h under salt treatment (Figure 7B).

## Discussion

To prevent accumulation of toxic Na^+^ amounts in the cytosol, active sodium efflux into the apoplast is one of the three strategies employed by plants to increase salt resistance (Greenway and Munns, 1980). Sodium ions are transported from the cytosol across the PM against the electrochemical gradient into the apoplast via Na^+^ /H^+^ antiporters. Secondary transporters in the PM of plant cells are activated by the PM proton pump, PM H^+^-ATPase, which is a major enzyme of the plant PM that plays a central role in physiological functions and adaptation of plants to changing conditions, especially stress. Thus, PM H^+^-ATPase may be important to environmental stress resistance in general (Palmgren, 2001; Palmgren and Nissen, 2011). Therefore, it can be assumed that PM H^+^-ATPases are controlled by multiple regulatory features that integrate signals from the environment. Our results were consistent with previous findings that saline conditions stimulate the hydrolytic activity of PM H^+^ -ATPase in root (Figure 1; Niu et al., 1996; Sibole et al., 2005). Moreover, in the present study, the enzyme responded rapidly, within 3 h, to salinity treatments. Therefore, rapid posttranslational modifications to PM H^+^-ATPase likely occurred in grape root under salt stress conditions. Posttranslational modification is the best-known mechanism for rapid regulation described to date, and involves the auto inhibitory action of the C-terminal domain of the enzyme (Hsu et al., 2009; Haruta et al., 2015). Further work will be needed to confirm whether phosphorylation of the C terminus of VvPMA occurs in grape root under salt stress.

In addition to posttranslational modifications, changes to PM H^+^-ATPase activity can partly result from changes in expression patterns of PM H^+^-ATPase genes. This enzyme is coded by a multigene family in grape, like in other plants (Figure 2; Arango et al., 2003). *VvPMA1* and *VvPMA3* (Figure 3) were the predominantly expressed isoforms found in grape root, and NaCl stress increased accumulation of their transcripts in the root (Figure 4). Moreover, the *VvPMA3* expression increased significantly faster than *VvPMA1* expression. These results suggested that transcription of *VvPMA3* responded more quickly than *VvPMA1* transcription in grape root under NaCl stress. Therefore, the root *VvPMA3* appeared to play a critical role in early resistance to salt stress.

More interestingly, however, retention of the second intron in *VvPMA1* mRNA was discovered under salt conditions (Figure 5A, Supplementary Figure 2). This intron retention produced a spliced isoform, VvPMA1β, shortened by 35 amino acids at the N-terminal domain (Supplementary Figure 3). Moreover, yeast complementation assays found that, compared with *VvPMA1*α, *VvPMA1*β enhanced the ability of *VvPMA1* to support yeast growth, at pH 5.5 and more acidic pH levels (Figures 6B,C). This finding confirmed earlier findings that small deletions from the N terminus *in vitro* increased the ability of the plant PM H^+^-ATPase to support yeast growth. Ekberg et al. (2010) showed that destabilization or complete removal of the N terminus of PM H^+^-ATPase somehow results in an unmasking of the extreme C terminus, which makes the penultimate threonine residue accessible for protein kinase-mediated phosphorylation and subsequent activation of the PM H^+^-ATPase. We have shown, for the first time, that removal of the N terminus of PM H^+^-ATPase can be achieved *in vivo* through AS.

Recent genome-wide gene expression analyses have suggested that mRNA splicing is under the control of developmental and stress signals (Seo et al., 2011; Marquez et al., 2012). Here, the results showed that although expression of *VvPMA1*α was higher than *VvPMA1*β expression in root, regardless of treatment (Figures 7A,B), the expression level of *VvPMA1*β still greatly increased under salt stress, suggesting that alternative splicing of *VvPMA1* was markedly induced under salt conditions. Previous results showed that splicing factors, such as serine/arginine-rich (SR), Ski-interacting protein (SKIP) etc., can control the AS of pre-mRNAs encoded by salt tolerance genes under salt stress in plants (Syed et al., 2012; Feng et al., 2015) and different growth conditions can modulate gene expression or protein modification of splicing factors causing dynamic changes in the splicing factor profile, further impacting expression of target genes. So we supposed that the expression of spliced variant *VvPMA1*β must be controlled by one or some specific splicing factors in grape root under salt conditions, moreover, *VvPMA1*β expression maintained low level in root under salt stress probably because the specific splicing factor has weak activity. Alternatively, PM H^+^-ATPase activity could be confined by an unknown feedback mechanism under different environment and overexpression of its gene might be disadvantageous to plant growth (Hashimoto-Sugimoto et al., 2013). Thus, it can be speculated that the amount of VvPMA1β protein with high activity is possible to be strictly controlled at posttranscriptional level in grape root under salt stress, to avoid wasting too much energy at feedback regulation of PM H^+^-ATPase activity. The specific splicing factor will be further screened from grape root and the function of *VvPMA1*β will be ascertained by transgenetic effectiveness in plant under salt stress.

Additionally, posttranscriptional regulation of *VvPMA1* in response to salt stress occurred at 3 h of treatment as early as transcriptional regulation of *VvPMA3* (Figures 4, 7). And both expression of *VvPMA1*β and *VvPMA3* reached peak before 24 h. Meanwhile *VvPMA1*α expression was unchanged markedly. After that, *VvPMA1*α expression obviously increased, while the other two diminished. Combining these findings with the dynamic change of the activity of PM H^+^-ATPase and VvPMA1β activity (Figure 1, Figure 6B), we predicted that *VvPMA1*β would have a positive effect on PM H^+^-ATPase activity in salt stress, albeit with phenotypic lag. Therefore, subtle changes in the ratio of intact VvPMA1 and the alternatively spliced variant would likely have profound effects on PM H^+^-ATPase activity in grape root under saline conditions in spite of changes to the quantity of PM H^+^-ATPase present. Further work is required to determine changes in the quantity of each PM H^+^-ATPase isoform under salt stress.

Taken together, the results of this study revealed that NaCl has a stimulatory effect on PM H^+^-ATPase in grape root. Increases to enzyme activity were accompanied by transcriptional increases. Furthermore, cloning and sequencing of the PCR-amplified fragments of PM H^+^-ATPase transcripts revealed the expression of an N-terminal truncated splice variant in response to NaCl treatment. This variant had a higher activity rate than the intact variant. These findings suggest that alternative splicing, in coordination with gene transcriptional control, may help to maintain the appropriate level of the PM H^+^-ATPase activity in grape roots under saline condition. Furthermore, it is also possible that differential regulation of the PM H^+^-ATPase isoforms within the same cell could be an important mechanism for responding to a diverse range of environmental fluctuations.

## Author contributions

NH, XZ, and HZ participated in the study conception and design. XJ contributed to RNA extraction and qPCR. NH, YD, and XH contributed to data analysis. NH wrote the manuscript. YD and XH critically revised the manuscript. All authors approved the final version of the manuscript.

### Conflict of interest statement

The authors declare that the research was conducted in the absence of any commercial or financial relationships that could be construed as a potential conflict of interest.

## Supplementary material

Supplementary Figure 1 **The relative expression levels of ***VvPMA2, VvPMA5***, and ***VvPMA7*** in leaf, stem and M-pericarp (mature pericarp) of grape**. *Vvactin* was used as an internal standard. The data were analyzed according to 2^−ΔCT^ method. The values are means ± SD (*n* = 5).

Supplementary Figure 2 **ClustalW alignment of the 5′-terminal region of ***VvPMA1***α and ***VvPMA1***β**. Gray line denoted start codon. Identical residues are shaded. Asterisks indicated highly conserved residues.

Supplementary Figure 3 **ClustalW alignment of VvPMA1α and VvPMA1β**. Identical residues are shaded. Amino acid residues not present within other sequences are denoted in dashes. Asterisks indicated highly conserved residues.

Supplementary Table 1 **Primer pairs for real-time PCR**.

Supplementary Table 2 **Primer pairs for VvPMA1**.
