# Supplementary figures and images for: Identification of a Novel Alternative Splicing Variant of VvPMA1 in Grape Root under Salinity
Source: Front Plant Sci. 2017 Apr 21;8:605. doi: 10.3389/fpls.2017.00605 (PMC5399082; doi:10.3389/fpls.2017.00605)

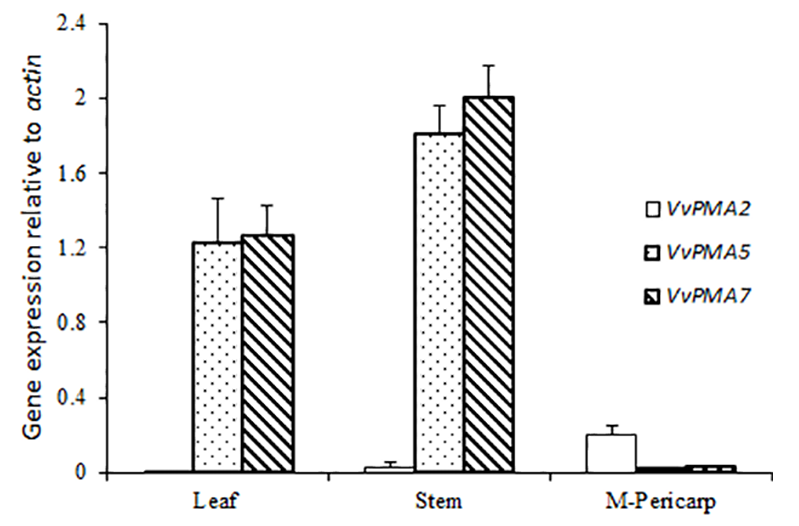

Supplement: Supplementary Figure 1 — The relative expression levels of VvPMA2, VvPMA5, and VvPMA7 in leaf, stem and M-pericarp (mature pericarp) of grape. Vvactin was used as an internal standard. The data were analyzed according to 2−ΔCT method. The values are means ± SD (n = 5). [file Image1.TIF]

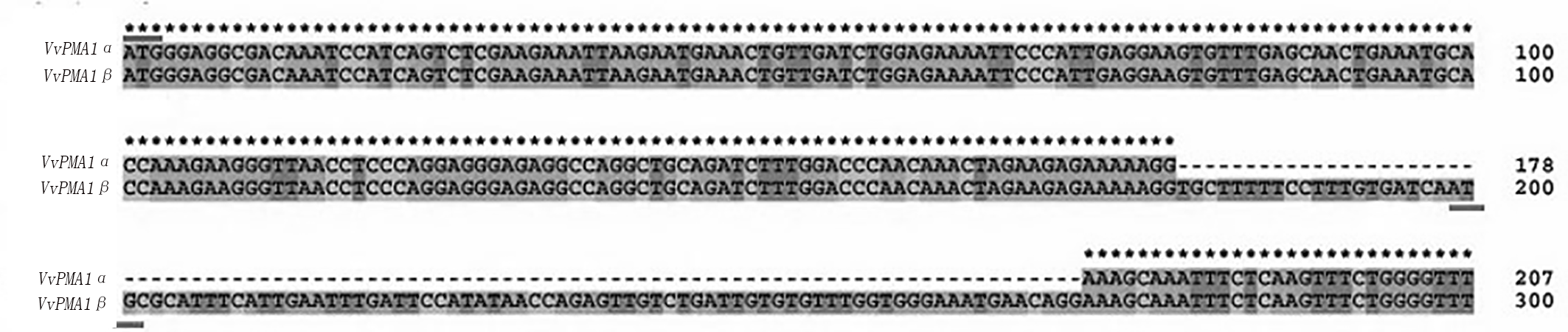

Supplement: Supplementary Figure 2 — ClustalW alignment of the 5′-terminal region of VvPMA1α and VvPMA1β. Gray line denoted start codon. Identical residues are shaded. Asterisks indicated highly conserved residues. [file Image2.TIF]

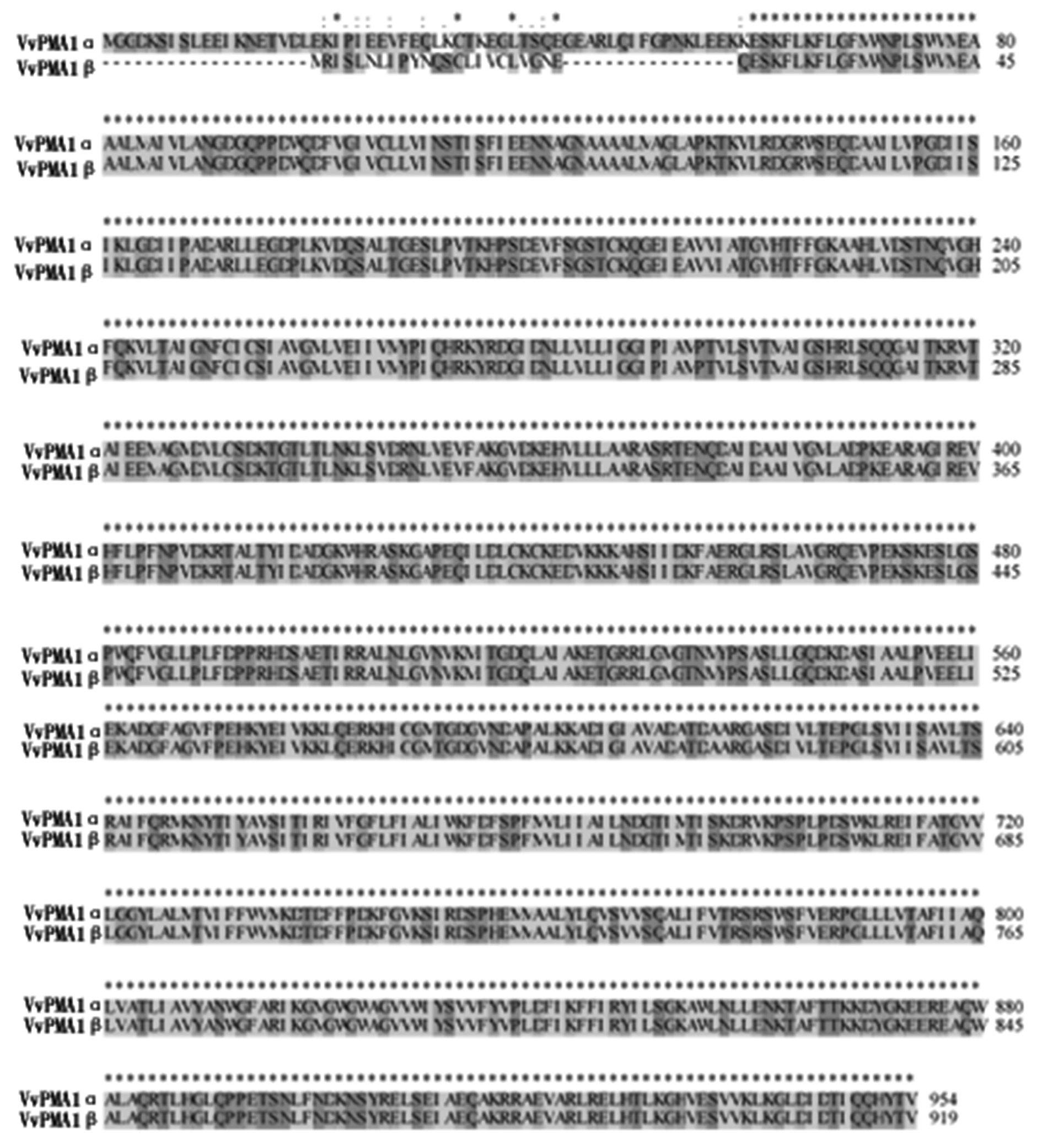

Supplement: Supplementary Figure 3 — ClustalW alignment of VvPMA1α and VvPMA1β. Identical residues are shaded. Amino acid residues not present within other sequences are denoted in dashes. Asterisks indicated highly conserved residues. [file Image3.TIF]
