# Supplementary material for: Identification of a Novel Alternative Splicing Variant of VvPMA1 in Grape Root under Salinity
Source: Front Plant Sci. 2017 Apr 21;8:605. doi: 10.3389/fpls.2017.00605 (PMC5399082; doi:10.3389/fpls.2017.00605)
Supplement: Supplementary Table 1 — Primer pairs for real-time PCR. [file Table1.DOCX]

**Supplementary Table 1 Primer pairs for real-time PCR**

| Gene name | Product length (bp) | primer(F) | primer(R) |
| --- | --- | --- | --- |
| *VvPMA1* | 169 | 5' GCTATTGAACAAGAAAAACCCG 3' | 5' GAAGAAGCTAAACACAACCCCT 3' |
| *VvPMA2* | 147 | 5' GGGACATTAACCTTATGGCAG 3' | 5' CAGTGTAGTGCGGATTGATGT 3' |
| *VvPMA2** | 104 | 5' CTTGGAGCTTACCTGGCCCT 3' | 5' TGGTGCTGATTGAAGTCAGGTA 3' |
| *VvPMA3* | 131 | 5' GAAAGAGAAGGAGAGAAACAAG 3' | 5' GGAAATAATAGACGGCAAAC 3 |
| *VvPMA4* | 134 | 5' GACCAAGATGTTCACTGACCAC 3' | 5' TCAGCCTCACCACGGATTC 3' |
| *VvPMA5* | 126 | 5' AAGGGACTGGACATTGAAACC 3' | 5' GCCCCATTTATGTATGTTTTGT 3’ |
| *VvPMA5** | 185 | 5' GCTACCGGGAGTTGTCTGAG 3' | 5' TCCTCACCTACACTCCAAACTTG 3' |
| *VvPMA6* | 183 | 5' TCACGTGGAATCAGTGGTGAG 3' | 5' GGCTGTCTGAAGGGTCACTC 3' |
| *VvPMA7* | 156 | 5' GAGGGCATTCAACAGCACTAC 3' | 5' TCCTTCCATCCTTCTATTCTCC 3' |
| *VvPMA7** | 182 | 5' CCAAGAGGCGTGCTGAAGTT 3' | 5' ACTTCCACTTTCCCTTTAGCCT 3' |
| *VvPMAα* | 120 | 5' TAGAAGAGAAAAAGGAAAGCA 3' | 5' CAACAAAGTCCTGCCAATC 3' |
| *VvPMAβ* | 228 | 5' GTTGTCTGATTGTGTGTTTGG 3' | 5' TGCCAGCATTGTTTTCTTC 3' |
| *Vvactin* | 134 | 5' GAGATTCCGTTGTCCAGAAGTC 3' | 5' CAATGTTGCCATAGAGGTCCTT 3' |

F denoted forward primers; R denoted reverse primers; * denoted another pair of primer of one gene.
