# Supplementary material for: Identification of a Novel Alternative Splicing Variant of VvPMA1 in Grape Root under Salinity
Source: Front Plant Sci. 2017 Apr 21;8:605. doi: 10.3389/fpls.2017.00605 (PMC5399082; doi:10.3389/fpls.2017.00605)
Supplement: Supplementary Table 2 — Primer pairs for VvPMA1. [file Table2.DOCX]

**Supplementary Table 2 Primer pairs for VvPMA1**

| Gene Name | Primer sequences |
| --- | --- |
| VvPMA1 | F: 5' CTCGAGAGATATGGGAGGCGACAAAT 3'  R: 5' GGGCCCTTCTTTCCCTTCTTGGTTCG 3' |
| VvPMA1α | F_1_: 5' TAGAAGAGAAAAAGGAAAGCA 3’  R : 5' CAACAAAGTCCTGCCAATC 3’ |
| VvPMA1β | F_2_: 5' GTTGTCTGATTGTGTGTTTGG 3'  R : 5' CAACAAAGTCCTGCCAATC 3’ |

F denoted forward primers; R denoted reverse primers; F with XhoI site and R with ApaI site (underline).
